# Element Pool Changes within a Scrub-Oak Ecosystem after 11 Years of Exposure to Elevated CO_2_

**DOI:** 10.1371/journal.pone.0064386

**Published:** 2013-05-23

**Authors:** Benjamin D. Duval, Paul Dijkstra, Bert G. Drake, Dale W. Johnson, Michael E. Ketterer, J. Patrick Megonigal, Bruce A. Hungate

**Affiliations:** 1 Department of Biological Sciences, Northern Arizona University, Flagstaff, Arizona, United States of America; 2 Merriam-Powell Center for Environmental Research, Northern Arizona University, Flagstaff, Arizona, United States of America; 3 Smithsonian Environmental Research Center, Edgewater, Maryland, United States of America; 4 Department of Natural Resources, University of Nevada-Reno, Reno, Nevada, United States of America; 5 Department of Chemistry and Biochemistry, Northern Arizona University, Flagstaff, Arizona, United States of America; DOE Pacific Northwest National Laboratory, United States of America

## Abstract

The effects of elevated CO_2_ on ecosystem element stocks are equivocal, in part because cumulative effects of CO_2_ on element pools are difficult to detect. We conducted a complete above and belowground inventory of non-nitrogen macro- and micronutrient stocks in a subtropical woodland exposed to twice-ambient CO_2_ concentrations for 11 years. We analyzed a suite of nutrient elements and metals important for nutrient cycling in soils to a depth of ∼2 m, in leaves and stems of the dominant oaks, in fine and coarse roots, and in litter. In conjunction with large biomass stimulation, elevated CO_2_ increased oak stem stocks of Na, Mg, P, K, V, Zn and Mo, and the aboveground pool of K and S. Elevated CO_2_ increased root pools of most elements, except Zn. CO_2_-stimulation of plant Ca was larger than the decline in the extractable Ca pool in soils, whereas for other elements, increased plant uptake matched the decline in the extractable pool in soil. We conclude that elevated CO_2_ caused a net transfer of a subset of nutrients from soil to plants, suggesting that ecosystems with a positive plant growth response under high CO_2_ will likely cause mobilization of elements from soil pools to plant biomass.

## Introduction

Many studies have evaluated the effects of elevated CO_2_ on nitrogen cycling, and focused on the hypothesis that tree growth response to elevated CO_2_ may be limited by N availability, or change with N use efficiency [1]–[4], but the impact of CO_2_ on elements other than N has been studied less frequently. The nutrients P, K and Ca can also limit plant productivity [5], [6], non-N nutrients can limit N_2_ fixation and C storage [7], and changes in Al, Mn and Fe concentrations might affect the availability of other mineral nutrients in soils [8]. To fully assess the impact of elevated CO_2_ on ecosystem nutrient cycling, it is important to evaluate effects on all elements that are necessary for plant nutrition and elements that control the availability of other nutrients in the soil system.

Photosynthesis and growth are often stimulated by elevated CO_2_ in C_3_ plants [9], [10], often leading to more biomass production. Increased growth increases nutrient demands [11]. It has been suggested that nutrients become more limiting for growth over time and can limit terrestrial C uptake [12]. Increased production of carbohydrates in plants is suggested to reduce element concentrations in plants [13]. Elevated CO_2_ generally reduces plant N concentration, but increased growth does not inherently dilute the concentration of other elements in plant tissues [14]–[18]. In sweet gum (*Liquidambar styraciflua*), Johnson et al. [17] found significant declines in foliar Fe concentration with elevated CO_2_. At the POP-EUROFACE CO_2_ experiment, there was no change in poplar leaf K or Ca concentrations, while Mg concentration actually increased in those trees [19]. A cross-experiment evaluation of elevated CO_2_ by Natali et al. [20] showed significantly lower Fe concentration in sweet gum at the Duke FACE site, decreased Al, V and Fe concentrations in sweet gum at the Oak Ridge FACE site, but increased Mn and Mo concentrations in *Quercus myrtifolia* at the Smithsonian Institution Elevated CO_2_ site in Florida. Lastly, a recent meta-analysis of 14 tree species and 10 nutrient elements found that elevated CO_2_ lowers Cu, Fe, K, Mg, P and S concentrations, but only at high N availability [15].

Element availability varies by soil type and ecosystem. Soil element availability is a function of soil organic matter content (SOM) and pH, with elements generally less adsorbed to metal oxides and SOM in acid soils [21]. Elevated CO_2_ has been shown to increase P availability, possibly a function of decline in SOM in the experimental plots [22]. Elevated CO_2_ has also been implicated in reducing leaching of soil N and P from upper soil layers [17]. A recent study found that trace metal concentrations increased in soils exposed to elevated CO_2_ at Duke FACE and Oak Ridge FACE, but decreased under elevated CO_2_ for every element surveyed at the Florida SERC experiment [20].

Johnson et al. [23] reported a decrease in foliar N and S concentration of scrub-oak (*Q. myrtifolia*), an increase in oak foliar Mn, and no change in P, K, Cu, B or Zn after 5 years of CO_2_ enrichment [23]. However, because biomass was significantly higher under elevated CO_2_, total plant pool sizes (on an area basis) for all elements were increased under elevated CO_2_. The data reported here were collected at the end of the Florida CO_2_ experiment, providing an assessment of the cumulative effect of more than a decade of CO_2_ enrichment on soil element pools. We evaluate the impacts of elevated CO2 on a scrub-oak stand in a fire-regenerated ecosystem by quantifying soil, litter and plant tissue (above and below ground tissue) element pools after 11 years, and to determine if elevated CO_2_ facilitated nutrient retention, loss or redistribution in this system.

Our overarching hypothesis was that declines in soil nutrients under elevated CO_2_ are quantitatively caused by increases in plant pools, in other words, that the cumulative impact of elevated CO_2_ is to redistribute elements in the plant-soil system. Specifically, we hypothesized that nutrient cycling in the Florida ecosystem changed under elevated CO_2_ in the following ways:

1) Increases in aboveground biomass driven by elevated CO_2_ will increase the pool size of elements in those tissues (leaves, stems, litter and roots).

2) Increased plant uptake depletes plant soluble element pools in soils exposed to elevated CO_2_.

3) Element retention in this ecosystem will increase under elevated CO_2_ because of increased plant element pools, especially in long-lived tissue like wood and coarse roots.

## Materials and Methods

### Study site

Our study was conducted at the Smithsonian Environmental Research Center’s long-term elevated CO_2_ experiment at Kennedy Space Center, Cape Canaveral, Florida, USA (28° 38′ N, 80° 42′ W). The experiment consisted of 16 octagonal open-top chambers that were 2.5 m high covering a ground surface area of 9.42 m^2^. Eight chambers were kept at ambient atmospheric CO_2_ concentration (ambient treatment) and 8 chambers were maintained at ambient +360 µmol mol^−1^ CO_2_ (elevated treatment) from May 1996 to June 2007. Soils at the site are acidic sands (Arenic Haplahumods and Spodic Quartzipsamments). The vegetation is Florida coastal scrub-oak palmetto [23], [24]. In the experimental chambers, greater than 90% of the aboveground biomass is scrub oak [25].

### Field collections

We harvested aboveground biomass in July 2007. We took foliar samples by collecting 5 fully expanded leaves per tree (*Q. myrtifolia*) from 5 distinct trees per plot. Oak stem samples were taken from 5 large branches from the main trunk per plot. We also took samples from the principal symbiotic nitrogen-fixing vine in the system, *Galactia elliottii*. We collected leaves from 5 *G. elliottii* vines per plot.

Soil was collected with a 7 cm diameter soil core at five locations within each chamber. We separated horizons as follows: A horizon (0–10 cm), E horizon (10–30 cm), E2 horizon (30–100 cm) and spodic horizon (Bh), a distinct zone of organic matter accumulation that varied from ∼100–250 cm depth. Because the spodic horizon varies in depth and is the deepest soil layer above the vadose zone, not all of the chambers were sampled to the same depth. The cores from each plot were combined per depth into a single composite sample for element analysis.

We collected litter from 1.18 m^2^ of the chamber. We collected roots from soil cores by sieving (2 mm mesh). For each soil depth, we separated roots by size into fine (<2 mm diameter) and coarse (>2 mm) fractions. Nutrient concentrations in root tissues were scaled up using root biomass estimates based on minirhizotron photographs (fine roots) and ground penetrating radar imaging [26].

### Sample Preparation and Element Analysis

We analyzed soil and plant tissues from the experimental site for the following elements (in order by atomic mass): Na, Mg, Al, P, S, K, Ca, V, Mn, Fe, Cu, Zn, Se, Sr and Mo. All glass wear and plastic containers used for sample extractions and digestions were acid washed in 0.5 M HCl for 48 hours prior to use. All acid reagents were of trace-metal clean purity. Prior to soil digestions and extractions, roots were removed from soil core samples and all soil was passed through a 2 mm sieve and oven dried at 105 °C. An acid digestion was used to prepare samples for measuring total soil element concentrations. Dried soil samples of 100–150 mg were ashed at 600 °C prior to acid digestion in a MARS microwave digestor. Microwave digestion was performed for 20 minute runs at 200 °C with trace metal grade, concentrated HF, HNO_3_^−^ and HCl, until all soil was dissolved into solution.

Plant-available element pools in soil were determined using an ammonium oxalate extraction [27]. One (1.0) g of soil was extracted in 15 ml of 0.3 M ammonium oxalate in a 60 ml sample cup, placed on a reciprocal shaker at 180 rpm for 18 h, filtered, diluted 10 times, and re-suspended in 10 ml of 0.32 M trace metal grade HNO_3_. We use this extraction as an estimate of plant-available element pools because the yields from this extraction fall within the published values for plant available elements and are consistent with the percent of the plant available element pool compared to the total (in this case acid digest) element pool [8], [28].

Plant tissues (leaves, woody biomass and roots) were oven dried at 60 °C for 24 h after collection. Roots were cleaned of excess soil by sonicating ∼3–5 g of root tissue in15 ml centrifuge tubes for 30 minutes in ultrapure (18 MΩ) water. The washed roots were again oven-dried for 24 h at 60°C. All plant samples were then ashed at 600 °C, and 500–600 mg of each ashed sample was acid digested on the MARS microwave digestor. Element analyses were conducted using Thermo X Series quadrupole ICP-MS.

Estimates of total plant biomass [25], were used to calculate the aboveground pool of elements. We multiplied element concentrations from *Q. myrtifolia* leaves and stems with total leaf and stem biomass to estimate the overall content of nutrients in the three oak species. *G. elliottii* element concentrations were likewise multiplied by biomass. The belowground plant element pool (to 100 cm) was calculated by multiplying the element concentration of roots by their biomass.

### Statistical Analyses & Data Availability

We analyzed the effects of CO_2_ on *Q. myrtifolia* biomass, element concentration, and element mass with a one-way ANOVA. We also used a two-way ANOVA model to test for CO_2_ effects and differences in the element mass of different plant pools (leaves, stems, litter and roots), and interactions between those factors. The effect of elevated CO_2_ on soil element mass was analyzed using a repeated measures ANOVA model with CO_2_ treatments and soil depth as the repeated measure. We employed the two-tailed Flinger-Killen test to check assumptions of equal variance [29]. Pairwise comparisons of CO_2_ effects on soil pools by depth were made using Tukey’s HSD test. Due to relatively low sample size (*n* = 8 per treatment), we use an alpha of 0.10 to determine significance [20].

To control for family-wise error rates, we used the False Discovery Rate (FDR) test to ensure that using a large number of pair-wise tests for CO_2_ effects did not yield a significant number of Type I errors. Our tests of multiple elements within a “group”, for example, soil plant available element pools, consisted of 15 individual ANOVAs. In all cases, the FDR expected less than one false discovery per group of multiple tests, justifying our use of multiple ANOVAs.

We also calculated the percent effect of elevated CO_2_ on nutrient pools:
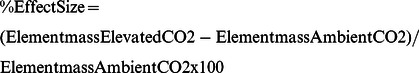



To ensure that we were able to detect differences due to the CO_2_ treatment with relatively low sample size, and reduce our study-level Type II error rate, we also used resampling with replacement to determine the % effect size of elevated CO_2_ effects on element pools, an approach complementary to ANOVA for determining differences between treatments [30]. We re-sampled from the sample population of element masses in elevated CO_2_ plots and ambient CO_2_ plots, with replacement (1000 iterations). This approach enabled us to determine the mean effect of elevated CO_2_ compared to the control plots, as well as calculate 90% confidence intervals around the mean effect size. We consider the CO_2_ effect meaningful if the confidence intervals do not overlap 0. Statistical analyses were performed in JMP, Microsoft Excel and R [31]. Data used in these analyses are available online via the University of Illinois’ Institute for Genomic Biology Public Data Archive [32].

## Results

The concentration of V and Ca decreased in *Q. myrtifolia* leaves exposed to elevated CO_2_ (F_1,14_ = 3.22, *P* = 0.09; F_1,14_ = 3.20, *P* = 0.09, respectively), but foliar S concentration increased (F_1,14_ = 5.66, *P* = 0.03). Elevated CO_2_ reduced the concentration of stem Ca (F_1,14_ = 5.93, *P* = 0.03), Mn (F_1,14_ = 3.17, *P* = 0.10) and Fe (F_1,14_ = 4.18, *P* = 0.06). Elevated CO_2_ did not significantly change the concentration of any element measured in root tissue or in the litter layer.

**Figure 1:**
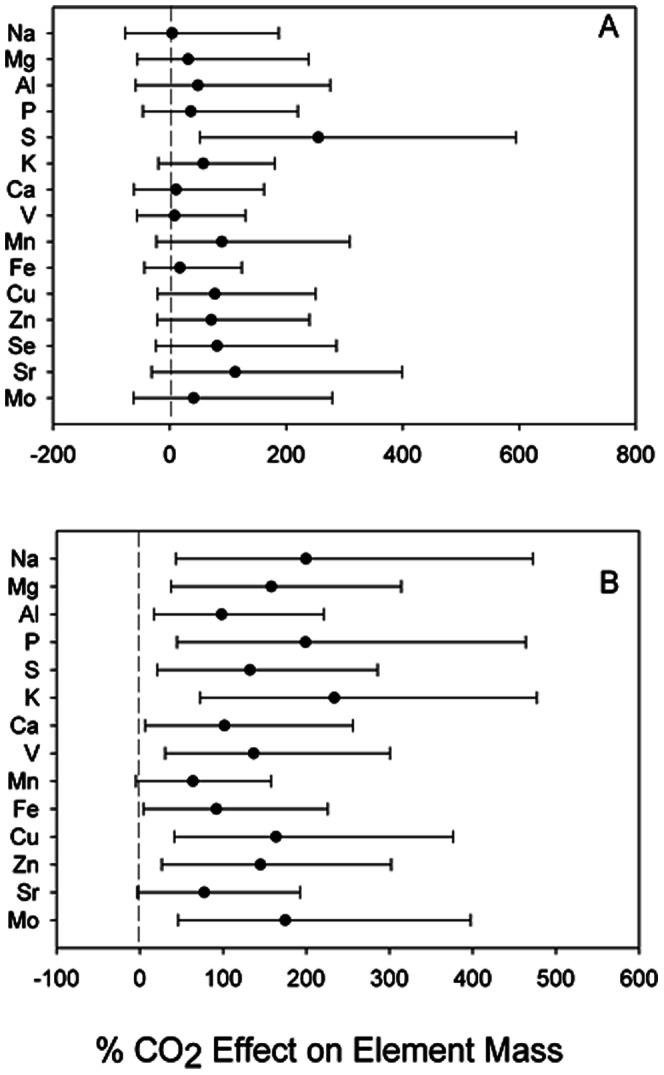
Re-sampled effect size (1000 iterations) of elevated CO_2_ compared to ambient CO_2_ means and 90% confidence intervals, for element pools in A) *Quercus* spp. leaves, B) *Quercus* spp. stems.

The total aboveground biomass of scrub oak exposed to elevated CO_2_ was ∼100% higher at the end of the experiment (F_1,14_ = 10.44, *P* = 0.01, from ref. 25). Pools of K and S in total above ground oak biomass were greater under elevated CO_2_ (*P*<0.05) compared to ambient CO_2_ (Table 1). The effect of CO_2_ exposure suggests a greater accumulation of S under elevated CO_2_ (Figure 1A). Oak stems under elevated CO_2_ hosted significantly greater pools of Na, Mg, P, K, V, Zn and Mo (ANOVA, *P*<0.10; Table 1), and our resample analysis suggests every element other than Mn and Sr was accumulated in woody tissue under elevated CO_2_ (Figure 1B).

**Table 1:** Element pools in the above ground plant biomass, litter layer and roots (0–100 cm, coarse + fine roots) under ambient and elevated atmospheric CO_2_, Kennedy Space Center, Florida.

Element	Tissue	Ambient CO_2_	SE	Elevated CO_2_	SE
Na (kg ^.^ ha^−1^)	Foliar	5.09	2.27	1.89	0.86
	Stems*	0.72	0.31	1.22	0.31
	Litter	45.51	6.60	42.54	6.55
	Roots to 100 cm*	87.45	4.54	146.81	26.50
Mg	Foliar	24.25	10.77	15.55	3.26
	Stems*	0.93	0.25	2.26	0.47
	Litter	32.62	4.87	35.75	5.40
	Roots to 100 cm*	69.81	4.63	97.90	12.17
Al	Foliar	0.16	0.08	0.11	0.02
	Stems	0.30	0.10	0.67	0.17
	Litter	41.60	6.51	47.21	6.40
	Roots to 100 cm	91.17	12.90	145.41	24.73
P	Foliar	12.45	4.55	9.52	1.80
	Stems*	0.02	0.01	0.07	0.02
	Litter	6.13	0.98	7.78	1.04
	Roots to 100 cm	3.04	0.29	4.07	0.56
S (g ^.^ ha^−1^)	Foliar	104.08	31.05	299.93	65.71
	Stems	6.26	1.90	13.54	3.11
	Litter	1184.07	157.57	1262.56	135.14
	Roots to 100 cm	168.09	20.35	419.00	130.25
K	Foliar	30.17	8.01	37.87	7.83
	Stems*	78.25	24.26	237.36	51.80
	Litter	80.34	11.40	95.38	14.71
	Roots to 100 cm	382.26	87.86	384.73	72.97
Ca	Foliar	79.76	36.18	48.52	9.10
	Stems	0.32	0.08	0.71	0.16
	Litter	16.96	2.89	20.22	2.53
	Roots to 100 cm	18.47	1.60	26.31	3.20
V (g ^.^ ha^−1^)	Foliar	0.44	0.18	0.30	0.06
	Stems*	7.57	3.51	20.81	6.96
	Litter	356.66	44.52	391.05	48.22
	Roots to 100 cm	0.47	0.07	1.04	0.25
Mn	Foliar	0.71	0.22	0.72	0.14
	Stems	0.07	0.02	0.14	0.03
	Litter	5.26	1.20	4.82	1.04
	Roots to 100 cm	1.98	0.39	2.62	0.53
Fe	Foliar	0.22	0.07	0.18	0.03
	Stems	0.09	0.04	0.13	0.04
	Litter	2.10	0.34	11.28	7.87
	Roots to 100 cm	1.34	0.22	1.74	0.24
Cu (g ^.^ ha^-1^)	Foliar	43.58	13.43	44.07	9.04
	Stems	7.08	2.00	16.06	3.95
	Litter	298.87	46.66	295.14	37.05
	Roots to 100 cm	678.74	118.14	1215.08	292.35
Zn	Foliar	0.20	0.07	0.22	0.04
	Stems*	0.05	0.01	0.09	0.02
	Litter	2.41	0.23	2.83	0.63
	Roots to 100 cm	2.20	0.18	4.37	1.10
Se (g ^.^ ha^−1^)	Foliar	0.14	0.03	0.16	0.06
	Stems	n/a	n/a	n/a	n/a
	Litter	n/a	n/a	n/a	n/a
	Roots to 100 cm	0.02	0.00	0.03	0.01
Sr (g ^.^ ha^−1^)	Foliar	235.23	124.75	173.36	41.78
	Stems	55.90	18.00	132.58	36.97
	Litter	2470.91	478.97	2431.55	282.15
	Roots to 100 cm*	3.70	0.43	4.40	0.58
Mo (g ^.^ ha^−1^)	Foliar	0.07	0.02	0.05	0.01
	Stems*	0.91	0.31	1.67	0.38
	Litter	241.24	28.68	264.25	31.25
	Roots to 100 cm	0.10	0.02	0.23	0.07

Asterisks denote significant ANOVA results for larger pools under elevated CO_2_ compared to ambient CO_2_ , ? denotes larger pools in ambient CO_2_ plots. All units are kg. ha-1 unless specified differently.

**Figure 2:**
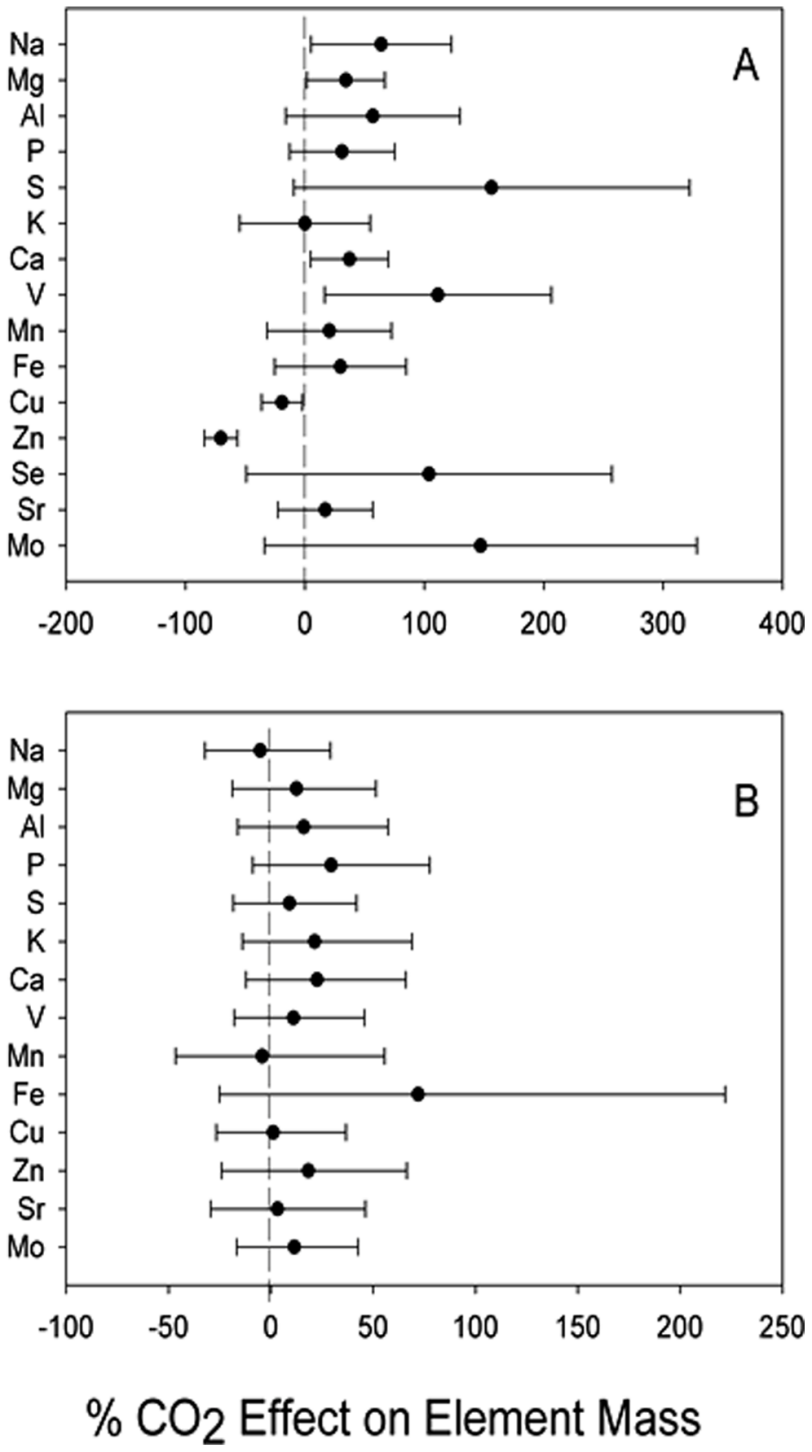
Re-sampled effect size (1000 iterations) of elevated CO_2_ compared to ambient CO_2_ means and 90% confidence intervals, for element pools in A) all plant roots to a depth of 1 m and B) the litter layer.

There was generally a positive effect of CO_2_ on the root element pool (Figure 1C). Indeed, our resample analysis showed significant CO_2_ effect on Na, Mg, Ca and V, and only a negative effect on Zn (Figure 2A). The mass of litter was not significantly changed by CO_2_ exposure, but compared to foliar and stem tissues there were large pools of Na, Mg, S, V, Fe, Cu, Sr and Mo in the litter layer (Table 1). There was at least a slight trend for a positive CO_2_ effect on litter pools for every element measured other than Na and Mn (Figure 2B).

Two-way ANOVA including CO_2_ treatment and plant pool as predictor variables revealed that there were significant differences among plant pools for every element measured. This difference was driven by larger pools of elements in roots than other plant material for every element other than Al and Ca, which were in larger quantity in leaf tissue (Table 1). There were significant, positive main CO_2_ effects on the overall above ground plant element pool for Ca (F_1,56_ = 2.76, *P* = 0.10), K (F_1,56_ = 2.99, *P* = 0.09) and Sr (F_1,56_ = 3.00, *P* = 0.08). There was also a significant CO_2_ by tissue pool interaction for those three elements (Ca, F_3,56_ = 2.77, *P* = 0.05; K, F_3,56_ = 3.00, *P* = 0.04; Sr, F_3,56_ = 3.09, *P* = 0.03).

**Figure 3:**
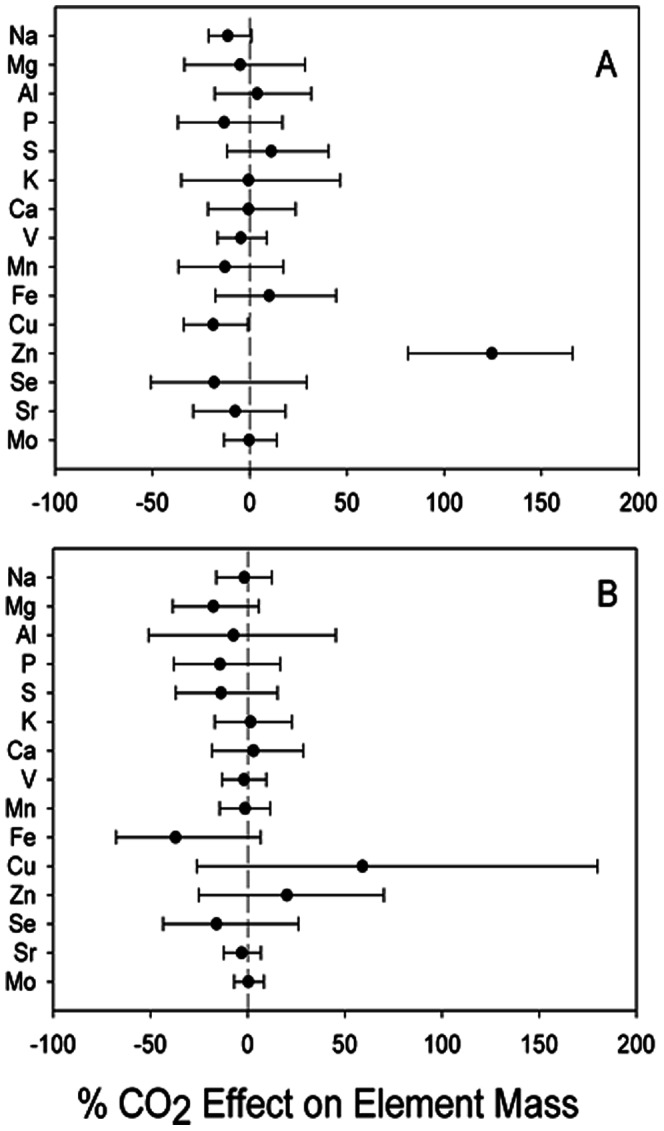
Re-sampled effect size (1000 iterations) of elevated CO_2_ compared to ambient CO_2_ means and 90% confidence intervals, for elements in A) total acid digest soil pool and B) plant available soil element pools.

Examining the entire soil profile, there was no CO_2_ effect on nutrient pools for the entire soil profile (0–100 cm + Bh) for either the total digest or plant available soil elements (Figure 3A, 3B). There were no CO_2_ effects when individual horizons were considered independently (Table 2, Table 3), other than plant available Al to a depth of 30 cm (F_1,29_ = 3.33, *P* = 0.08). However, we observed a significant effect of horizon depth for all total digest elements, and a horizon effect for plant-available nutrient pools of Mg, Al, K, Ca, V, Mn, Se and Sr (Table 3). We also observed trends in the pool size for different elements at different soil depths. Irrespective of CO_2_ treatment, there was a large total (acid digest) pool of K in the E2 and Bh (spodic) horizons, and this held for plant available K as well (Table 2, Table 3). Consistent with other spodosols, a large pool of Fe was found in the 10–30 cm portion of the horizon (Table 2). Plant available molybdenum was found in the greatest quantity in the deeper parts of the profile (Table 3).

**Table 2:** Total pool (acid digest) of soil elements after 11 years exposure to under ambient and elevated atmospheric CO_2_, Kennedy Space Center, Florida elevated CO_2_, Kennedy Space Center.

Element	Soil Horizon	Ambient CO_2_	SEM	Elevated CO_2_	SEM
Na (kg ^.^ ha^−1^)	A (0–10 cm)	340.5	23.6	287.2	19.9
	E (10–30 cm)	845.6	128.9	756.4	69.2
	E2 (30–100 cm)	231.1	11.1	216.1	13.4
	Spodic	44.6	2.8	53.4	10.7
Mg (kg ^.^ ha^−1^)	A (0–10 cm)	46.3	3.1	36.7	1.9
	E (10–30 cm)	64.8	6.6	69	12.8
	E2 (30–100 cm)	7.5	1.5	8.7	1.8
	Spodic	1.5	0.2	1.6	0.2
Al (kg ^.^ ha^−1^)	A (0–10 cm)	1687.9	219.9	1284.5	145.3
	E (10–30 cm)	2523.9	501.2	2524	366.6
	E2 (30–100 cm)	515.7	122.9	995.6	545.1
	Spodic	245.6	35.2	247.5	39
P (kg ^.^ ha^−1^)	A (0–10 cm)	9.8	2.2	9.1	2
	E (10–30 cm)	131.8	25.9	103.1	16
	E2 (30–100 cm)	10	3.6	13	3.5
	Spodic	4.1	0.7	5	0.9
S (kg ^.^ ha^−1^)	A (0–10 cm)	n/a			
	E (10–30 cm)	n/a			
	E2 (30–100 cm)	6.6	1.4	7.6	1.2
	Spodic	1.2	0	1.2	0
K (kg ^.^ ha^−1^)	A (0–10 cm)	680.3	62.4	536.7	49.3
	E (10–30 cm)	1527.1	320.1	1346.8	146.5
	E2 (30–100 cm)	126462.7	35285.6	117669.6	31055.1
	Spodic	50398	5619.8	41336.6	4337.6
Ca (kg ^.^ ha^−1^)	A (0–10 cm)	30.7	1.6	26.5	1.4
	E (10–30 cm)	65.4	6.2	63.9	3.9
	E2 (30–100 cm)	17.2	5.1	18.4	4.7
	Spodic	4.8	0.1	5	0.2
V (kg ^.^ ha^−1^)	A (0–10 cm)	2.50	0.18	1.99	0.13
	E (10–30 cm)	5.01	0.63	4.87	0.60
	E2 (30–100 cm)	2.44	0.20	2.42	0.17
	Spodic	0.48	0.02	0.52	0.03
Mn (kg ^.^ ha^−1^)	A (0–10 cm)	19.5	1.5	15.3	1
	E (10–30 cm)	45.8	9.9	40.3	7.9
	E2 (30–100 cm)	4.3	0.8	4.1	0.6
	Spodic	0.8	0	0.9	0.1
Fe (kg ^.^ ha^−1^)	A (0–10 cm)	72.7	5.8	56.1	3.9
	E (10–30 cm)	137.2	25.5	140	25
	E2 (30–100 cm)	21.9	7.2	19.7	4.5
	Spodic	n/a			
Cu (kg ^.^ ha^−1^)	A (0–10 cm)	3.06	0.20	3.11	0.36
	E (10–30 cm)	6.23	0.50	5.47	0.39
	E2 (30–100 cm)	1.94	0.08	1.97	0.07
	Spodic	316.8	9.3	315.7	10
Zn (kg ^.^ ha^−1^)	A (0–10 cm)	10.3	1.2	8.1	1
	E (10–30 cm)	16.7	1.5	15.2	1.2
	E2 (30–100 cm)	3.2	0.2	3.7	0.3
	Spodic	0.5	0	0.5	0
Se (g ^.^ ha^−1^)	A (0–10 cm)	150.9	6.4	143.4	6.1
	E (10–30 cm)	337	37.4	339.7	21.2
	E2 (30–100 cm)	276.3	264.9	45.3	206.1
	Spodic	54.9	6.5	55.5	8.1
Sr (kg ^.^ ha^−1^)	A (0–10 cm)	6.89	0.64	4.93	0.39
	E (10–30 cm)	11.25	2.45	11.20	1.55
	E2 (30–100 cm)	3.24	0.52	3.01	0.45
	Spodic	1.03	0.16	1555.3	0.43
Mo (g ^.^ ha^−1^)	A (0–10 cm)	318.2	20.4	289.9	12.9
	E (10–30 cm)	685.7	34.1	681.9	30.4
	E2 (30–100 cm)	1785.3	17.4	1758.9	34.6
	Spodic	307.5	6.2	316.9	9

**Table 3:** Pool of plant available (ammonium oxalate extractable) elements after 11 years exposure to under ambient and elevated atmospheric CO_2_, Kennedy Space Center, Florida elevated CO_2_, Kennedy Space Center.

Element	Soil Horizon	Ambient CO_2_	SEM	Elevated CO_2_	SEM
Na (kg ^.^ ha^−1^)	A (0–10 cm)	5.12	0.94	4.79	0.65
	E (10–30 cm)	15.20	1.10	15.09	2.15
	E2 (30–100 cm)	11.64	0.29	11.14	0.15
	Spodic	11.94	0.20	10.81	0.60
Mg (kg ^.^ ha^−1^)	A (0–10 cm)	8.51	2.13	5.41	0.87
	E (10–30 cm)	7.61	1.03	7.04	1.00
	E2 (30–100 cm)	2.50	0.33	2.22	0.12
	Spodic	4.60	0.19	3.54	0.57
Al (kg ^.^ ha^−1^)	A (0–10 cm)	8.61	1.81	5.25	1.90
	E (10–30 cm)	37.95	11.95	14.42	2.45
	E2 (30–100 cm)	312.01	73.30	286.22	85.62
	Spodic	67.92	2.80	53.47	7.65
P (kg ^.^ ha^−1^)	A (0–10 cm)	3.50	0.77	3.24	0.70
	E (10–30 cm)	46.99	9.25	36.77	5.69
	E2 (30–100 cm)	7.41	1.55	7.38	1.88
	Spodic	4.81	0.11	3.67	0.77
S (kg ^.^ ha^−1^)	A (0–10 cm)	12.03	2.67	11.14	2.39
	E (10–30 cm)	161.66	31.81	126.50	19.56
	E2 (30–100 cm)	25.49	5.32	25.39	6.47
	Spodic	16.57	0.37	12.62	2.65
K (kg ^.^ ha^−1^)	A (0–10 cm)	8.20	1.74	7.04	1.06
	E (10–30 cm)	13.98	3.28	10.06	1.33
	E2 (30–100 cm)	5725.41	422.47	5996.16	389.53
	Spodic	18408.19	605.24	14427.58	2228.08
Ca (kg ^.^ ha^−1^)	A (0–10 cm)	3.12	1.17	3.11	1.03
	E (10–30 cm)	38.78	3.93	39.55	3.47
	E2 (30–100 cm)	0.63	0.03	0.64	0.03
	Spodic	1.30	0.01	1.01	0.19
V (g ^.^ ha^−1^)	A (0–10 cm)	32.97	6.24	14.00	3.05
	E (10–30 cm)	20.98	3.27	15.77	1.05
	E2 (30-100 cm)	152.86	8.68	157.44	12.85
	Spodic	149.04	2.74	133.07	8.72
Mn (g ^.^ ha^−1^)	A (0–10 cm)	122.37	19.94	93.03	11.27
	E (10–30 cm)	141.58	11.82	148.75	19.07
	E2 (30–100 cm)	283.02	11.80	281.44	7.11
	Spodic	282.94	8.98	239.83	22.77
Fe (kg ^.^ ha^−1^)	A (0–10 cm)	7.60	2.16	4.42	1.78
	E (10–30 cm)	27.59	10.71	9.56	1.79
	E2 (30–100 cm)	14.76	3.55	11.53	3.83
	Spodic	3.62	0.17	2.93	0.36
Cu (kg ^.^ ha^−1^)	A (0–10 cm)	2.26	0.73	2.64	0.86
	E (10–30 cm)	6.12	1.52	10.66	4.05
	E2 (30–100 cm)	0.32	0.08	0.31	0.08
	Spodic	0.34	0.01	0.29	0.03
Zn (kg ^.^ ha^−1^)	A (0–10 cm)	0.58	0.15	0.47	0.12
	E (10–30 cm)	0.97	0.19	1.30	0.32
	E2 (30–100 cm)	0.76	0.17	0.94	0.18
	Spodic	0.87	0.03	0.73	0.07
Se (g ^.^ ha^−1^)	A (0–10 cm)	1.00	0.27	0.65	0.22
	E (10–30 cm)	5.07	2.19	1.74	0.37
	E2 (30–100 cm)	n/a			
	Spodic	177.24	12.07	n/a	
Sr (g ^.^ ha^−1^)	A (0–10 cm)	31.63	3.55	27.64	4.19
	E (10–30 cm)	85.51	11.01	78.84	10.80
	E2 (30–100 cm)	168.69	7.72	164.63	6.23
	Spodic	142.55	2.77	128.73	6.97
Mo (g ^.^ ha^−1^)	A (0–10 cm)	0.99	0.68	0.21	0.02
	E (10–30 cm)	1.25	0.54	0.49	0.03
	E2 (30–100 cm)	97.09	1.11	96.18	1.46
	Spodic	82.78	0.65	80.41	1.19

**Figure 4:**
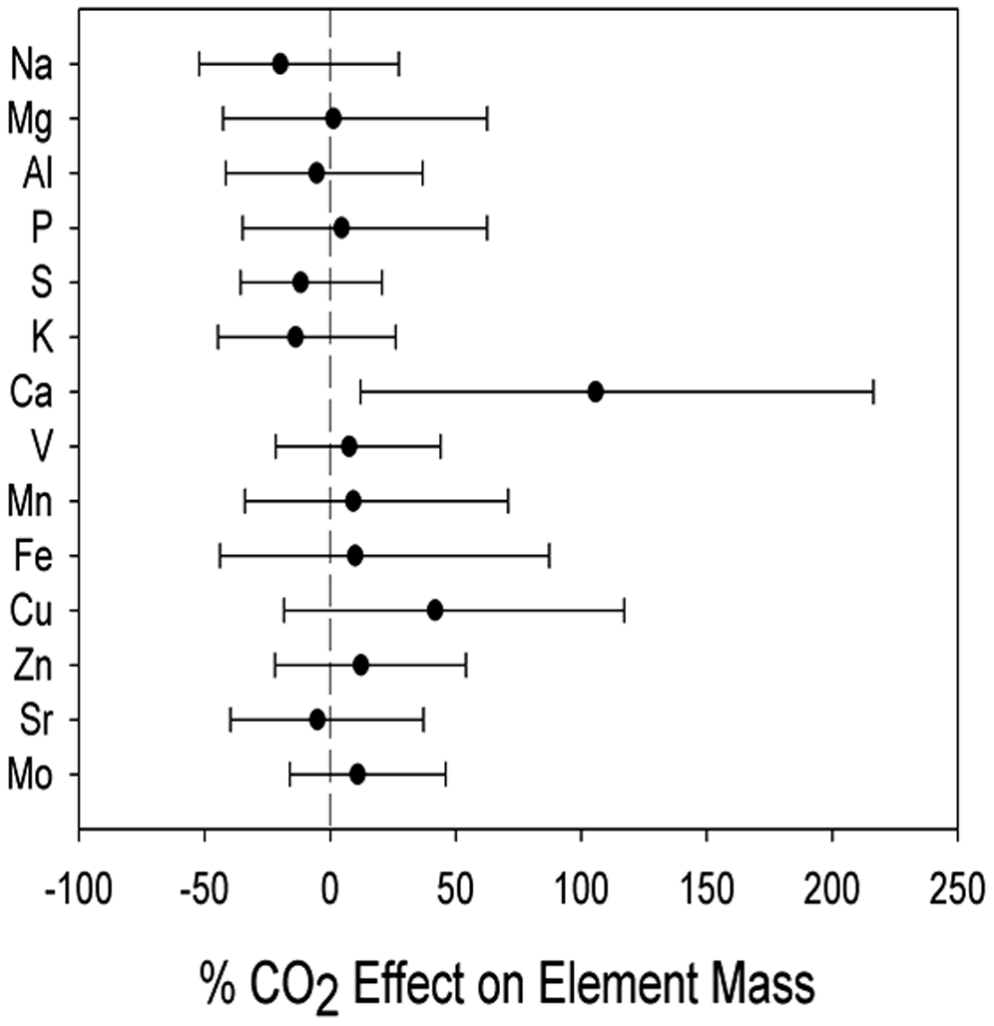
Re-sampled effect size (1000 iterations) of elevated CO_2_ compared to ambient CO_2_ means and 90% confidence intervals, for the total ecosystem element pool, calculated as the difference between the total plant and plant available element pools in soil under elevated CO_2_ compared to ambient CO_2_.

The effect of CO_2_ on the total ecosystem element pool, calculated as the sum of the total plant and plant available element pools in soil, was only significantly higher under elevated CO_2_ for Ca (Figure 4).

## Discussion

While elevated CO_2_ is expected to lower element concentrations in plant leaves [13], element concentrations in oak leaves we measured after 11 years of elevated CO_2_ exposure were generally not significantly impacted by high CO_2_. Calcium and V concentrations decreased, but S concentrations were higher in oak leaves in the elevated CO_2_ treatment. Scrub oaks at the Florida experiment showed significantly greater growth over the 11 years they were exposed to elevated CO_2_ [25]. The increased above ground oak biomass under elevated CO_2_ was high enough to consistently lead to increases in above ground plant and litter nutrient element pools irrespective of changes in element concentration (Table 1, Figure 1, Figure 2B).

We did not measure significant differences in soil element pools under elevated CO_2_ compared to ambient conditions. Combined with the sustained biomass stimulation of oaks under elevated CO_2_, there is no evidence that non-nitrogen nutrients are limiting growth after extended CO_2_ enrichment. There was a strong signal for a positive effect of elevated CO_2_ on root nutrient pools (Figure 1C), and the overall pool of elements in roots for oaks exposed to both elevated and ambient CO_2_ was often orders of magnitude higher than the element content of stems and leaves, driven by the large below ground biomass pool [33]. Thus, it is possible that these oaks are liberating nutrient elements from the total element pools into soluble forms by increasing production of root exudates, facilitating mycorrhizal colonization and changes to rhizosphere chemistry that facilitate nutrient uptake [34]–[37]. At the end of our study, root biomass was significantly higher under elevated CO_2_ treatments [33]. If oaks under elevated CO_2_ are shifting C allocation belowground, which is in turn providing greater root surface area and potentially more root exudate production, it would explain both the possibility that oaks are mining the soil for elements to meet their nutritional demands, as well as the positive effect of CO_2_ on root element pools (Figure 2B).

We do not have direct measurements of *Q. myrtifolia* mining soils for nutrients via root exudation and rhizosphere acidification. However, a source of nutrient liberation (and therefore facilitation of movement from soils to plant biomass) could come from the “priming” effect of elevated CO_2_ on organic matter mineralization, which could enhance the release of nutrients like P, Ca and metals bound to SOM. Indeed, we observed that the increase in Ca stocks in oaks was higher than the decline of extractable Ca in the soils (Figure 4). This phenomenon has been observed for N at this site, in the form of increased N mineralization under elevated CO_2_ [38], and the Ca result supports the hypothesis that CO_2_ induced soil priming increases nutrient availability could be a general phenomenon.

Calculating the CO_2_ effect of total ecosystem elements showed that CO_2_ enhances Ca retention but not significantly so for other elements (Figure 4). Because of the fire regime of this system, non-volatile elements sequestered in plant biomass will eventually return to the soil, but elements leached from the soil system to the water table are effectively gone from the system [39].

Liu et al. [40] measured increased leaching of Mg (385%), K (223%), Ca (167%) and NO_3_^−^ (108%) under elevated CO_2_, and attributed element loss to accelerated mineral weathering and higher soil water content under elevated CO_2_. Element loss through leaching is permanent, and we expect that soluble forms of elements that migrate downward through the soil profile will be exported from the system via lateral transfer [28]. However, the total pool of most elements is large relative to the plant available pool (Table 2, Table 3), and soluble forms of elements (especially K, Fe and Mg) can also be replenished in the soil via geochemical processes like chemical weathering, which may be accelerated by exposure to elevated CO_2_ [34], [40]. Indeed, our observed trend for lower concentrations of amorphous Al-oxides under elevated CO_2_ at the Florida site could be a result of accelerated chemical weathering [41].

Our results demonstrate that nutrient cycling is substantially altered after 11 years of exposure to elevated CO_2_, but the CO_2_ effect is element dependent [15], [42]. The strong, positive growth response of oaks to CO_2_ [25] led to increased pools of some elements (Na, V, Zn and Mo) in plant biomass and quantifiably lower plant available pools of most elements throughout the soil profile (Table 3). However, because there were only significant changes in the movement of some elements, it is likely that CO_2_ effects on element cycles are not easily generalized.

## Conclusions

Our results support the hypothesis that increases in oak biomass under elevated CO_2_ would increase the pool of nutrient elements in oak tissues. We also observed measurably lower stocks of most nutrients in soils under elevated CO_2_. The observation that Ca was retained in this system under elevated CO_2_ opens the possibility that some plants actively mine soils under elevated CO_2_ for nutrients other than N.
